# Getting Your Sea Legs

**DOI:** 10.1371/journal.pone.0066949

**Published:** 2013-06-19

**Authors:** Thomas A. Stoffregen, Fu-Chen Chen, Manuel Varlet, Cristina Alcantara, Benoît G. Bardy

**Affiliations:** 1 Affordance Perception-Action Laboratory, University of Minnesota, Minneapolis, Minnesota, United States of America; 2 Department of Recreation Sport & Health Promotion, National Pingtung University of Science and Technology, Pingung, Taiwan; 3 Movement to Health Laboratory, EuroMov, University of Montpellier-1, Montpellier, France; 4 Escola de Educação Física e Esporte, Universidade de São Paulo, São Paulo, Brazil; 5 Movement to Health Laboratory, EuroMov, University of Montpellier-1, Montpellier, France; 6 Institut Universitaire de France, Paris, France; McMaster University, Canada

## Abstract

Sea travel mandates changes in the control of the body. The process by which we adapt bodily control to life at sea is known as *getting one's sea legs*. We conducted the first experimental study of bodily control as maritime novices adapted to motion of a ship at sea. We evaluated postural activity (stance width, stance angle, and the kinematics of body sway) before and during a sea voyage. In addition, we evaluated the role of the visible horizon in the control of body sway. Finally, we related data on postural activity to two subjective experiences that are associated with sea travel; seasickness, and mal de debarquement. Our results revealed rapid changes in postural activity among novices at sea. Before the beginning of the voyage, the temporal dynamics of body sway differed among participants as a function of their (subsequent) severity of seasickness. Body sway measured at sea differed among participants as a function of their (subsequent) experience of mal de debarquement. We discuss implications of these results for general theories of the perception and control of bodily orientation, for the etiology of motion sickness, and for general phenomena of perceptual-motor adaptation and learning.

## Introduction

Stable control of the body is fundamental to successful interaction with the environment [1]. Body motion affects – either positively or negatively – our ability to maintain efficient and effective interactions with our surroundings. In healthy adults, stable control of bodily orientation is routine, but challenges to the control of bodily orientation can bring about global changes in behavior. Examples include changes in gait and overall movement patterns following stroke [2], and in the frail elderly [3]. Therefore, while theories of the perception and control of bodily orientation are important for basic science (e.g., for understanding relations between perception and action, and the embodiment of cognition in bodily activity) such theories are important also for clinical applications (e.g., for predicting and preventing falls in the elderly).

### Theories of orientation

A common assumption is that bodily orientation should be controlled relative to the direction of gravity [4], [5]. The fact that we routinely control orientation on vehicles (e.g., cars, aircraft, ships) is not widely regarded as having implications for general theories. Stance on moving laboratory devices typically has been used as a model for stance on stationary surfaces, rather than being treated as a subject of interest in its own right; an example is moving platform posturography [6]. Some scholars have argued that gravity is not a fundamental referent for the control of bodily orientation [7], [8]. Differentiation between the two types of theories has been hampered by the paucity of quantitative data about the control of bodily orientation on vehicles. Researchers who study human performance on vehicles (and vehicle simulators) understand that such research can have implications for general theories of perceptual-motor control [9], [10]. However, research on vehicles typically has been limited by several factors. Vehicles that are used for research can be prohibitively expensive (e.g., spacecraft, research aircraft); the same is true of motion-base vehicle simulators [9]. In addition, the duration of exposure to vehicle motion often is not sufficient to permit researchers to observe adaptation. Finally, research vehicles (and vehicle simulators) typically allow for very small samples sizes, and often are limited to unusual populations, such as astronauts or aircrew.

Greenwald [11] noted that innovations in experimental methods sometimes lead to qualitative changes in theoretical understanding [12]. Developments in motion sensing technology now enable scientists to measure directly the quantitative kinematics of human movement on vehicles. Using this technology, we created a method for the study of dynamic body orientation on ships at sea. We used this method to develop new insights into general (i.e., theoretical) aspects of bodily control. A central question in our study was how visible referents in the environment would affect dynamic bodily orientation, how these effects might change as participants adapted to ship motion, and how this adaptation process might affect other aspects of the more global adaptation process.

### Perceptual-motor adaptation and learning

Our method emerges from new technologies combined with an ancient adaptation stimulus. When a traveler embarks on a sea voyage the onset of ship motion is discrete, the presence of ship motion is continuous over many hours, days, or weeks, and adaptation is obligatory. On ships at sea it is possible to use contemporary technologies to monitor movement kinematics in relatively large samples of participants as they progress through the adaptation process. In the present study we asked how control of the body would change when maritime novices embarked on a sea voyage, and how properties of bodily control would be related to some of the subjective experiences that are associated with sea travel.

A ship at sea is in constant motion, including both linear and angular components relating to three axes of motion. Linear motion consists of surge (fore-aft), sway (lateral), and heave (vertical). Angular motion consists of roll, pitch, and yaw, which are rotations around the surge, sway, and heave axes, respectively. The resulting motions typically are complex and are difficult to simulate with motion-base devices. Simulators are also limited in terms of the duration of exposure, which rarely exceeds several hours. We were able to assess postural activity and subjective experiences as novice mariners were exposed continuously to ship motion.

### Controlling the body at sea

Sea travel predates the written word. Archeologists have recovered remains of ships up to 8000 years old [13] while other evidence suggests that human seafaring began no later than 30,000 years ago and may extend back 60,000 years [14]. By comparison, wheeled vehicles and equine domestication are less than 6000 years old. Thus, watercraft may be the earliest form of vehicular travel. Sea travel remains important in contemporary life. Each year, more than 10 million people take vacation cruises from North America alone [15]. Thus, the need to perceive and control stance relative to ships is not only one of the most ancient constraints on human movement but also one of the most persistent, with ongoing relevance.

Life at sea is characterized by control of the body on a moving surface. Stabilizing the body relative to a moving ship requires control actions different from those used on land. Qualitatively, these changes are well known to mariners, and are the subject of anecdotal accounts over many centuries. Recently, body sway at sea has been evaluated in controlled experimental research [16], [17], [18]. These studies have focused on experienced mariners; typically, working crewmembers with many years of maritime experience. In maritime novices the transition from land to sea entails a period of adaptation during which we learn to control the body relative to the moving support surface. This process, known as *getting your sea legs*, can last anywhere from a few minutes to several days [19].

Nautical lore is rich in anecdotes about how people get their sea legs but these anecdotes have not been subjected to empirical evaluation in experimental research. We conducted the first experiments relating the process of getting one's sea legs to quantitative data on control of the body. In designing our experiments we selected independent and dependent variables that have been widely studied in research on land, and we adapted these to phenomena that are associated with the process of getting one's sea legs. We used a fully within-participants design: The same individuals participated in all four experiments. This integrated approach provides new insights into the nature of adaptation to life at sea, but also has implications for general theories of the perception and control of the body.

Research on ships at sea is not a substitute for laboratory research. Rather, it provides an important complement to laboratory studies. In the laboratory we can manipulate parameters of stimulus motion [20], which are not under experimental control at sea. Conversely, in the laboratory it is difficult, inconvenient, and expensive to expose large numbers of research participants to stimulus motion over long periods of time, which makes it very difficult to study long-term adaptation and learning. At sea, extended exposure for large numbers of participants is convenient; indeed, it is routine. Unlike other vehicles, such as automobiles, aircraft, and spacecraft, ships offer large numbers of research participants who are exposed to the same stimulus motion continuously over long periods of time and can be studied at minimal expense. As a novel method for the study of perceptual-motor adaptation, research on ships at sea can offer new windows into understanding of general theoretical issues [11] as well as applications both at sea and on land.

### Summary

In an integrated series of experiments carried out with participants from a single voyage, we addressed several aspects of the process of getting one's sea legs. To evaluate the general influence of ship motion we took measurements before the voyage began (i.e., when the ship was at the dock), and again each day the ship was at sea. In Experiment 1 we evaluated changes in foot positioning that were related to the transition from land to sea. In Experiment 2 we evaluated changes in body sway that were related to the transition from land to sea, with a focus on the role of the visible horizon. In Experiment 3 we evaluated relations between foot positioning and body sway, on the one hand, and the severity of seasickness, on the other. In Experiment 4 we evaluated relations between body sway and mal de debarquement.

## General Method and Background Data

### Ethics Statement

The experimental protocol, #0711S21081, was approved in advance by the University of Minnesota IRB and informed consent was obtained from each participant in writing.

Our study was conducted as part of the Spring 2012 voyage of the Semester at Sea, an academic program operated by the Institute for Shipboard Education. The experiments were carried out during the first week of the voyage. Approximately 500 undergraduates from dozens of colleges and universities participated in the voyage, which began at Nassau, the Bahamas. After leaving Nassau the ship was at sea for two days before arriving at Roseau, in the Commonwealth of Dominica, where it remained for two days. Before the voyage began we collected data while the ship was tied up at the dock (January 19; Day 0). The ship was scheduled to depart Nassau in the evening on January 19 but due to a last-minute schedule change actually departed on January 20, in the evening. We then collected data on each of the first two full days at sea (January 21 and 22; Day 1 and Day 2, respectively). All data were collected on board the ship.

Students were not permitted to bring alcohol or illegal drugs onto the ship, and were subject to search when boarding the ship in any port. In addition, cabins were subject to random, unannounced searches at any time. Students found in violation of the policies on alcohol and drugs were ejected from the program at the next port of call and sent home. Alcohol was not served to students during the first week of the voyage.

### Participants

A total of 40 individuals participated, ranging in age from 19 to 28 years. Three people participated only on Day 0 and, consequently, were not included in any of our analyses. For the 37 remaining participants the mean age was 20.68 years, mean height was 176.27 cm, and the mean weight was 65.30 kg.

### Apparatus and Experimental Setting

The research was conducted on board the M/V *Explorer*, which was 180 meters long with a 26-meter beam. The ship displaced 25,000 tons and cruised at 24 knots. The experiments were conducted on the aft end of deck 4, an open space approximately 20 m wide by 10 m deep. A safety railing surrounded the perimeter; otherwise, the area provided an unimpeded view of the ocean from the ship's stern.

Data on the ship's motion were collected using the accelerometer in a MacBook Pro laptop computer running SeisMac [21]. We recorded data on linear acceleration along three axes, with each axis sampled at 25 Hz. The accelerometer was not sensitive to angular acceleration.

### Procedure

Each day we collected data from 08∶00–12∶00 and from 12∶30–16∶30. In Nassau (Day 0), participants were recruited as they boarded the ship. Volunteers reported to deck 4 aft throughout the day, at their own convenience. On the first and second full days at sea (Day 1 and Day 2, respectively), participants reported for testing at their own convenience. Some individuals who participated on Day 0 did not return on subsequent days. In addition, due to technical failures data on body sway were unusable in some cases. For these reasons there are small differences in the number of participants across experiments.

The informed consent procedure was completed before testing on Day 0. Also on Day 0 participants completed a questionnaire about their motion history. One a 1–4 scale (1 =  Much, 2 =  Some, 3 =  Little, 4 =  None), participants' mean reported experience at sea was 2.37 (some). On a 1–5 scale (1 =  Always, 2 =  Frequently, 3 =  Sometimes, 4 =  Rarely, 5 =  Never), participants' mean reported frequency of seasickness was 3.97 (rarely). On a 1–5 scale (1 =  Extremely, 2 =  Very, 3 =  Moderately, 4 =  Slightly, 5 =  Not At All), participants' mean reported general susceptibility to motion sickness was 3.72 (moderate). On each day of postural testing participants completed the Motion Sickness Questionnaire (see Experiment 3), after which they removed their shoes. We then measured stance width, stance angle, and standing body sway.

### Ship motion

The dock in Nassau was within a breakwater, such that ship motion at the dock was negligible, and power spectra were flat. At sea, the weather generally was clear, with light winds. On Day 2, there were isolated squalls of rain. On the Beaufort scale [22], the sea state was 4 on Day 1, and 5 on Day 2. On Day 1 the peak frequencies were 0.14 Hz, 0.85 Hz, and 0.13 Hz for linear acceleration along the surge, sway, and heave axes, respectively, with power at these peak frequencies ranging from −20 to −40 dB. On Day 2, the peak frequencies were 0.17 Hz, 0.95 Hz, and 0.17 Hz for linear acceleration along the surge, sway, and heave axes, respectively, with power ranging from −20 to −30 dB.

## Experiment 1

In a laboratory study McIlroy and Maki [23] measured foot positioning when 262 participants (healthy adults) were asked to stand quietly with their feet positioned comfortably. Stance width was defined as the distance between the midline of the heels, and the mean stance width was 17.0 cm (SD  = 4.0 cm). Stance angle was defined as the angle between the feet, and the observed mean was 15.1° (SD  = 11.5°). These results demonstrate both variability and consistency in preferred foot positioning during terrestrial stance.

Stoffregen, Chen, Yu, and Villard [24] evaluated experienced mariners. On land, stance width and angle were comparable to means reported by McIlroy and Maki [23]. At sea, stance angle did not differ from land, but mariners significantly increased their stance width. In Experiment 1, we asked whether maritime novices would alter stance width or angle at sea. We measured foot positioning when before the voyage, and on each of the first two days at sea.

## Method

### Participants

Seven males and 23 females participated on all three days of the experiment and so were included in our analysis. For these 30 individuals the mean age was 20.63 years (SD  = 2.04 years), and the mean height was 167.89 cm (SD  = 8.16 cm).

### Procedure

Stance width was measured twice each day, once with the participant facing forward (i.e., toward the bow) and once facing port (i.e., athwartship). The method was the same as used by Stoffregen, Chen, Yu, and Villard [24]. The experimenter stood approximately 3 m in front of the participant and asked him or her to take three steps forward and then stop. Using a tape measure, we measured the distance between the midline of the heels (stance width) and the distance between the great toes.

### Data Analysis

We evaluated stance width in terms of the distance between the midlines of the heels. We evaluated stance angle in terms of the ratio of the distances between the heels and the great toes. We conducted separate repeated measures ANOVAs on stance width and stance angle with factors Days (0, 1, 2) and orientation (facing bow vs. facing athwart).

## Results

**Figure 1:**
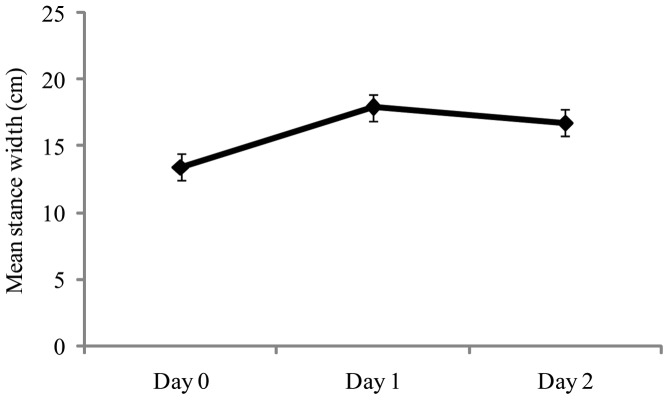
Experiment 1: Mean stance width (the distance between the midline of the heels) as a function of days. The figure illustrates the statistically significant effect of days. The error bars represent standard error of the mean.

Stance width results are summarized in Figure 1. The main effect of Days was significant, F(2,58)  = 19.78, *p*<.001, partial η^2^  = 0.405. Post-hoc tests revealed that Day 0< Day 1 =  Day 2. There were no other significant effects. Our analysis of stance angle yielded no significant effects. The heel-toe ratio did not change from land to sea (Day 0 mean  = 0.964; Day 1 mean  = 0.995; Day 2 mean  = 1.149), or as a function of orientation (bow mean  = 1.17, athwart mean  = 0.975). The interaction also was not significant.

## Discussion

In maritime novices we measured stance width and angle before a sea voyage and during the first two full days at sea. Before the beginning of the voyage mean stance width was within one standard deviation of the value reported by McIlroy and Maki [23] for young adults assessed in a terrestrial setting. Accordingly, our sample was representative. Novice mariners increased their stance width in response to ship motion: At sea, stance width was significantly greater than at the dock, and did not change across days at sea. These results indicate that the increase in stance width was completed during the initial hours of the voyage.

Stance angle did not differ between testing at the dock and testing at sea. Thus, participants increased the distance between their feet but not the angle between them. The selective adjustment of stance width reflects effects seen in experienced mariners: Stoffregen, Chen, Yu, & Villard [24] also found that the transition from land to sea influenced stance width but not stance angle. Our participants were not instructed to increase their stance width, either by the experimenters or by the Semester at Sea program. Thus, the adjustment was self-generated. The increase in stance width seems to have been an adaptive, self-selected change in body configuration in response to the experience of ship motion.

It would be interesting to determine how quickly novice mariners increase their stance width. Does the change occur in the first few hours at sea, or in the first few minutes? Is the adjustment related to the amount of time spent standing, or would it occur equally among people who were seated or reclining at the beginning of a voyage?

## Experiment 2

Anecdotal reports suggest that, at sea, bodily stability can be improved by standing on the open deck of a ship and looking at the horizon. Many authorities advise maritime novices to adopt this strategy, including cruise companies [25]; clinical otologists [26]; general medical reference Web sites [27]; and scholarly analyses of human performance at sea [19]. This advice contrasts qualitatively with phenomena reported on land. On land, body sway exhibits what we call the *Grand Canyon effect*, in which the magnitude of body sway is inversely related to the distance of visual targets. When we look at nearby targets (e.g., within arm's reach) body sway tends to be small. When we look at more distant targets body sway tends to be greater [28], [29], [30]. The logical limit of the effect occurs when the visual target is the horizon, for example, when standing at the edge of the Grand Canyon. Bles, Kapteyn, Brandt, and Arnold [31] contrasted sway in the laboratory (when participants looked at a target that was 0.5 m distant) with sway when participants stood on balconies of a building. On a balcony 20 m above the ground the distance to the horizon was 25 m. At this height, sway in the body's mediolateral (ML) axis was greater than in the lab.

Mayo et al. [17] measured standing body sway in experienced mariners on land and at sea. On the dock immediately before a voyage, participants looked at a nearby target (0.4 m in front of them) or at a distant mountain ridge. In this setting experienced mariners exhibited the classical Grand Canyon effect, with reduced body sway when viewing a nearby target and increased sway when viewing the horizon. The same individuals were later tested at sea using three visual targets. The near target (distance  = 0.4 m) and the mid-distance target (distance  = 3.0 m) were on the ship. The third target was the horizon. When viewing targets on the ship the magnitude of postural sway increased with target distance; the Grand Canyon effect. However, looking at the horizon was associated with a decrease in the amount of body sway, relative to sway when viewing the mid-distance target.

In Experiment 2, we asked a similar question in the context of maritime novices. We measured standing body sway as participants looked at nearby targets and at the horizon (unlike Mayo et al. [17], we did not include a mid-distance target). We collected data when the ship was at the dock and during each of the first two days at sea. Prior to beginning a voyage, we predicted that maritime novices would exhibit the Grand Canyon effect, with greater body sway when viewing the horizon, relative to sway when viewing a nearby target. In addition, we predicted that maritime novices would exhibit a reversal in this relation during the first 48 hours of a sea voyage.

Previous studies relating body sway to the distance of visual targets have examined only measures of spatial magnitude [17], [28], [29], [30]. In Experiment 2 we included a measure of spatial magnitude (the positional variability of the center of pressure), but also a measure of the temporal dynamics of sway. The temporal dynamics of sway can be influenced by variations in the difficulty of visual tasks [32], [33], and we asked whether this would be true also for variations in target distance.

## Method

### Participants

We analyzed body sway data from six males and 22 females who participated on all three days of the experiment. For these 28 individuals the mean age was 20.64 years (SD  = 2.08 years) and the mean height was 167.86 m (SD  = 8.11 cm).

### Apparatus

Data on body sway were collected using two force plates. One was a laboratory device (AccuSwayPlus, AMTI, Watertown, MA), which was controlled by a laptop computer running Balance Clinic software. The other was a Nintendo Wii Balance Board, (WBB), which was controlled through a Bluetooth wireless connection by a laptop computer running a custom software application [32]. The WBB has been validated for use in scientific studies of standing body sway [34], [35], [36]. On each device we sampled the position of the center of pressure (COP) in the antero-posterior (AP) and mediolateral (ML) axes, and stored the data on disk for later analysis. The AMTI was sampled at 50 Hz, and the WBB was sampled at 32 Hz. Following previous studies with the AMTI [37] and the WBB [32] we did not filter the data.

During postural testing the near target was the head of a photographic tripod that was 10 cm tall and 6 cm wide and had irregular surface indentations [17]. The tripod was placed atop small tables. Supporting the head on the tripod allowed for the head to be placed and removed quickly. Consistent placement was ensured by setting the tripod legs at marked positions on the tables, and the table legs at marked positions on the deck. The tripod was adjusted so that the head was at eye height for each participant. The nearby target was present only in the near target condition. The tripod was absent when the visual target was the visible horizon.

### Procedure

**Figure 2:**
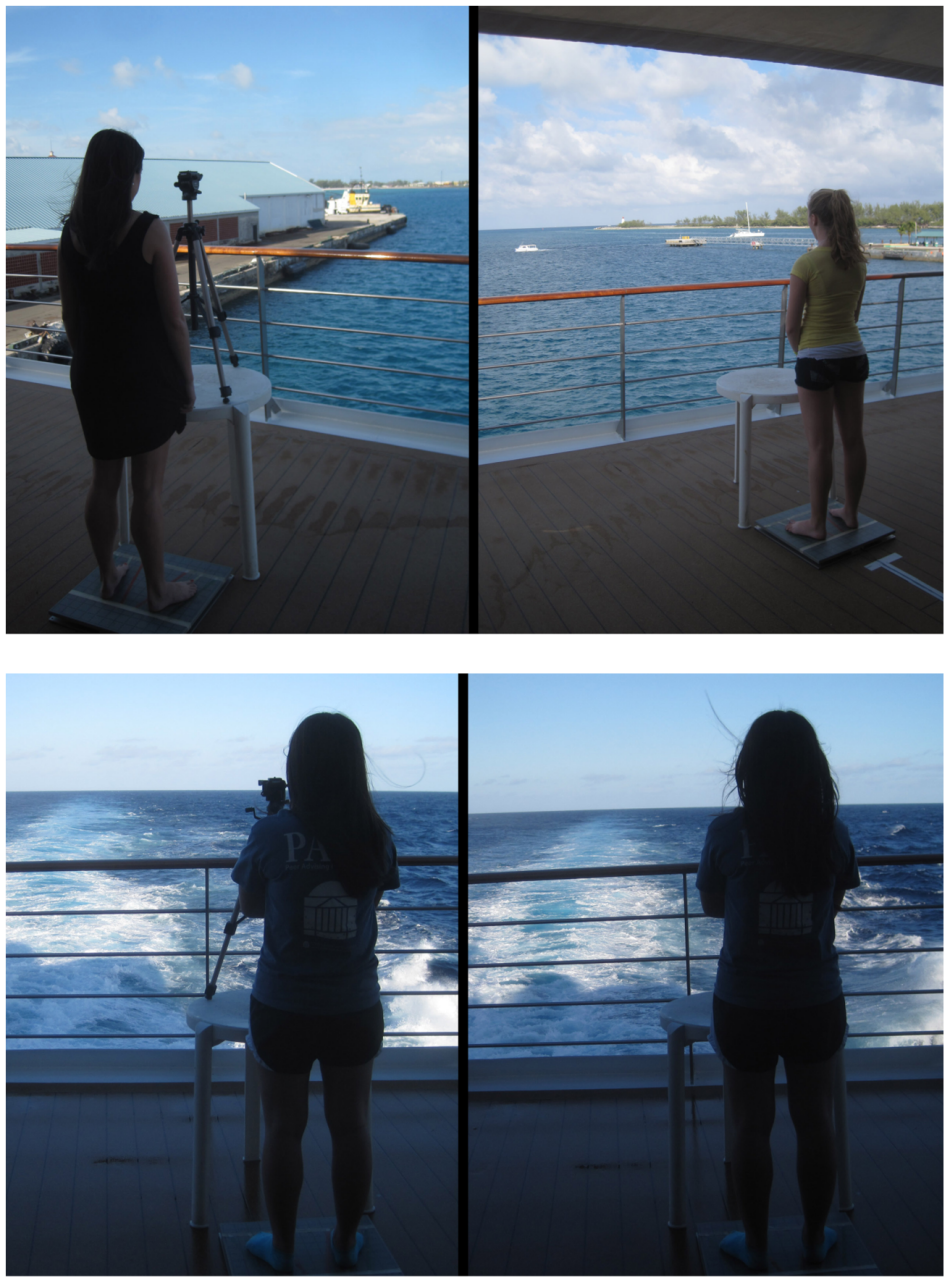
Setting and conditions for body sway testing. A. Viewing of the nearby target and the horizon at the dock. B. Viewing of the nearby target and the horizon at sea.

The experimental setting is illustrated in Figure 2. During measurement of body sway participants stood with torso perpendicular to the ship's long axis. The line of gaze was parallel with the ship's long axis, directed toward the stern. The Accusway and WBB force plates were set up on the open deck, approximately 3 m apart with different experimenters operating each device. With this arrangement we could collect data from two participants simultaneously, which permitted us to run an adequate number of participants each day. Each participant stood on only one device each day. Individuals were not required to stand on the same force plate across days.

Participants stood with their heels on a line near the back of the force plate. On each force plate, lateral foot placement was determined by a pair of lines on the plate, such that the heels were 17 cm apart (the mean value for self-selected stance width on land [23]). The lines were at an angle of 10° relative to each other, so that stance angle was held constant. Participants were not instructed to minimize sway, but rather were told to stand comfortably. Participants could hold their arms in any position (e.g., at sides, with hands clasped, or crossed; Figure 2), but were instructed not to move their arms during trials. Immediately before each trial each force plate was calibrated with the participant standing off the plate. Each trial lasted 60 s. There were a total of 6 trials per participant per day; three with the near target and three looking at the horizon. On each day, the order of target distance conditions was counterbalanced across participants. In the near condition, the tripod was positioned so that the near target was 50 cm in front of the heels.

### Data analysis

We separately analyzed the spatial magnitude and temporal dynamics of body sway. We assessed spatial magnitude in terms of the positional variability of the COP, which we operationalized as the standard deviation of position. We assessed the temporal dynamics of movement using detrended fluctuation analysis, or *DFA*. DFA describes the relation between the magnitude of fluctuations in postural motion and the time scale over which those fluctuations are measured [38]. DFA has been used in several studies of the control of stance [39], and in our own research at sea [16],[40]. We conducted inferential tests on α, the scaling exponent of DFA, which was analyzed separately for movement in the AP and ML axes. The scaling exponent is an index of long-range autocorrelation in the data, that is, the extent to which the data are self-similar over different time scales. White noise, which is uncorrelated, yields α  = .5. The presence of long-range autocorrelation is indicated by α >.5. Pink noise (also known as 1/f noise) is indicated when α  = 1.0. Values of α >1.0 indicate nonstationary activity that resembles a random walk, while α >1.5 indicates Brownian noise. On land, quiet stance in healthy adults tends to be nonstationary, typically yielding 1.0> α >1.5. We have found similar results on ships at seas [16], [40]. We did not integrate the time series before conducting DFA.

We conducted separate repeated measures analyses of variance (ANOVA) on positional variability of the COP and on α of DFA. For each analysis the factors were days (Day 0, Day 1, Day 2), target distance (nearby target vs. horizon), and axis (AP vs. ML), with repeated measures on the days and target distance factors. We defined AP and ML movement relative to the force plate, such that the AP plane was parallel to the line of sight and the ML plane was normal to the line of sight.

## Results

**Figure 3:**
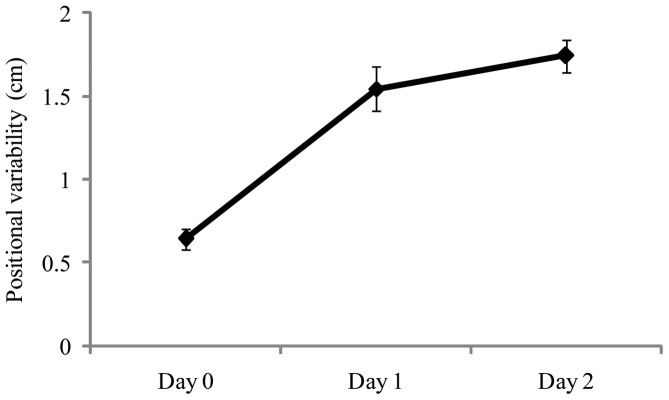
Experiment 2: Mean positional variability of the COP as a function of days. The figure illustrates the statistically significant effect of days. The error bars represent standard error of the mean.

**Figure 4:**
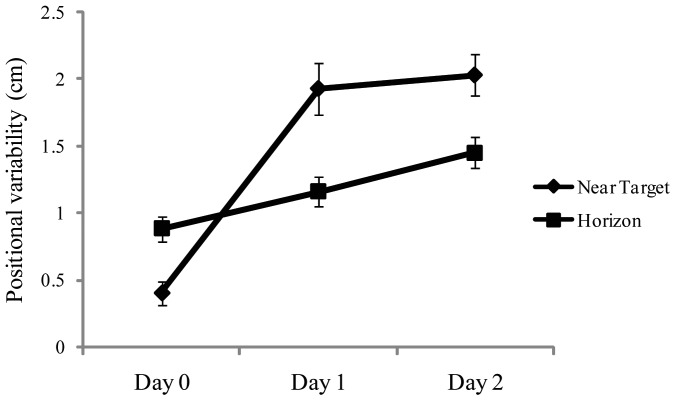
Experiment 2: Mean positional variability of the COP during viewing of the nearby target and the horizon, as a function of days. The figure illustrates the statistically significant interaction between target distance (nearby target vs. horizon) and days. The error bars represent standard error of the mean.

**Figure 5:**
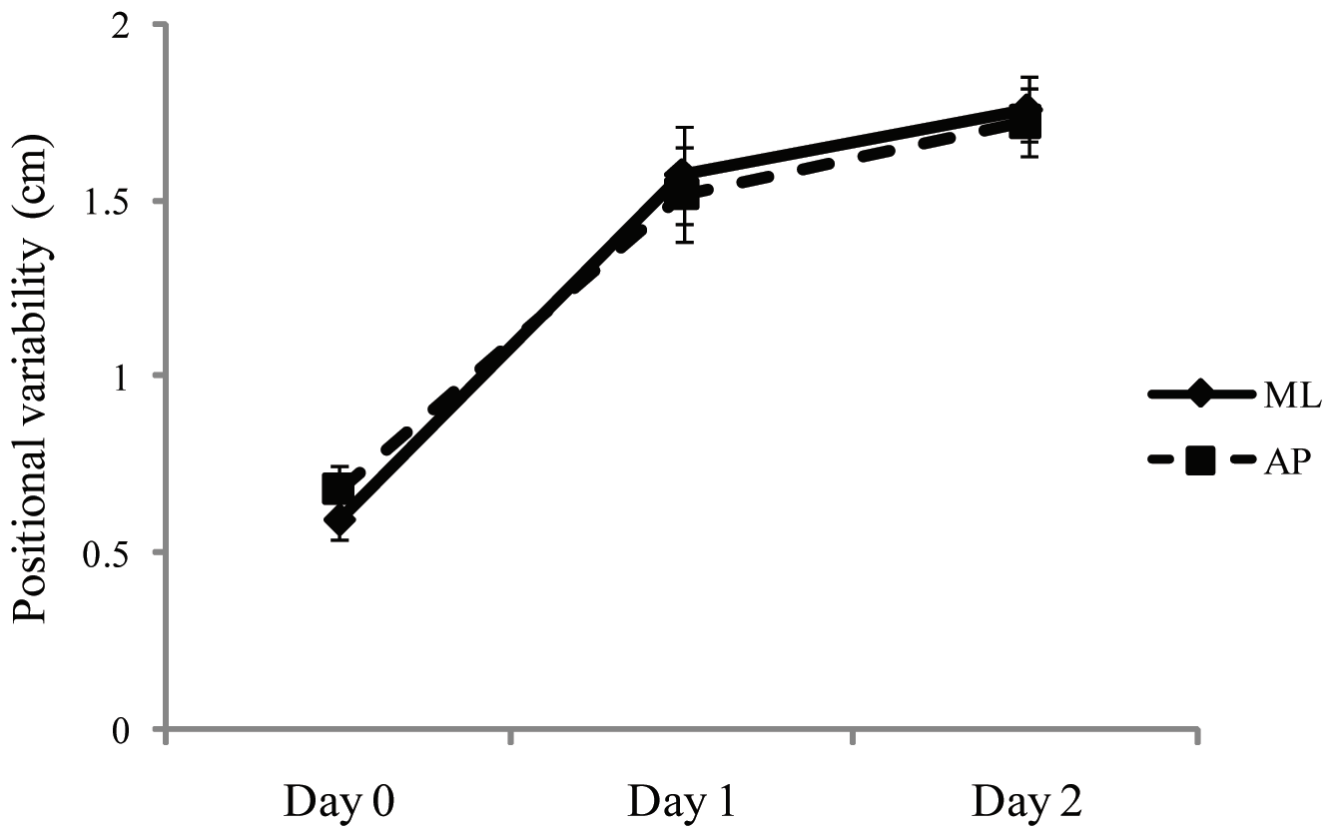
Experiment 2: Mean positional variability of the COP for the AP and ML axes, as a function of days. The figure illustrates the statistically significant interaction between axes and days. The error bars represent standard error of the mean.

For the positional variability of the COP, ANOVA revealed a significant main effect of days, F(1,28)  = 48.75, *p*<.001, partial η^2^  = 0.635, which is illustrated in Figure 3. Post hoc tests revealed that Day 0< Day 1 =  Day 2. Consistent with previous studies of experienced maritime crewmembers [17], [18] our novice participants swayed more at sea than when the ship was at the dock. The main effect of target distance conditions was also significant, F(2,56)  = 6.66, *p*<.015, partial η^2^  = 0.192. Sway was greater when looking at the nearby target (mean  = 1.47, SD  = 0.13), than when looking at the horizon (mean  = 1.16, SD  = 0.42). There was a significant interaction between the days and target distance factors, F(2,28)  = 37.96, *p*<.001, partial η^2^  = 0.576, which is illustrated in Figure 4. At the dock (Day 0), positional variability was greater when looking at the horizon than when looking at the near target, replicating classical effects [31]. At sea, positional variability was greater when looking at the near target. Finally, the day × axis interaction was significant, F(2,56)  = 10.84, *p*<.001, partial η^2^  = 0.279 (Figure 5).

**Figure 6:**
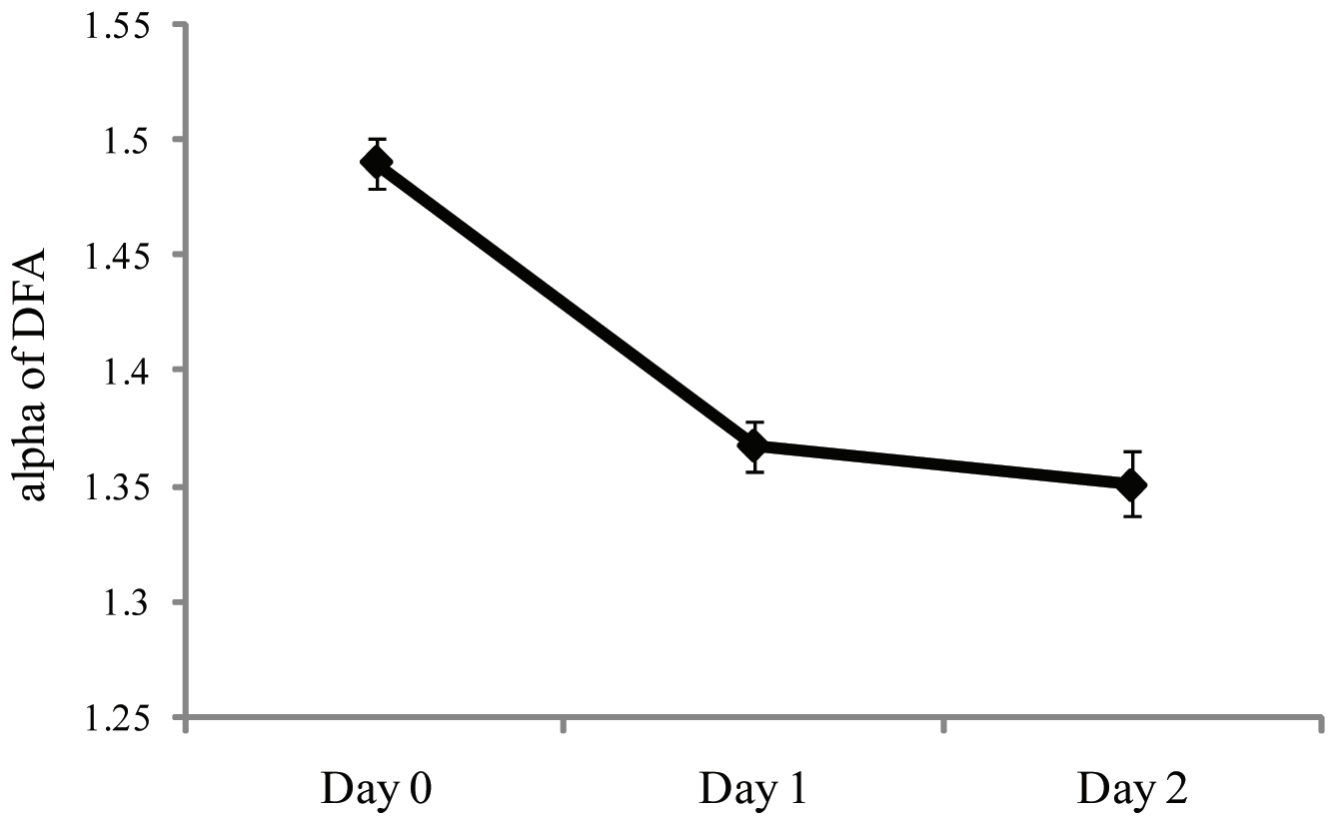
Experiment 2: Meanα of DFA as a function of days. The figure illustrates the statistically significant effect of days. The error bars represent standard error of the mean.

**Figure 7:**
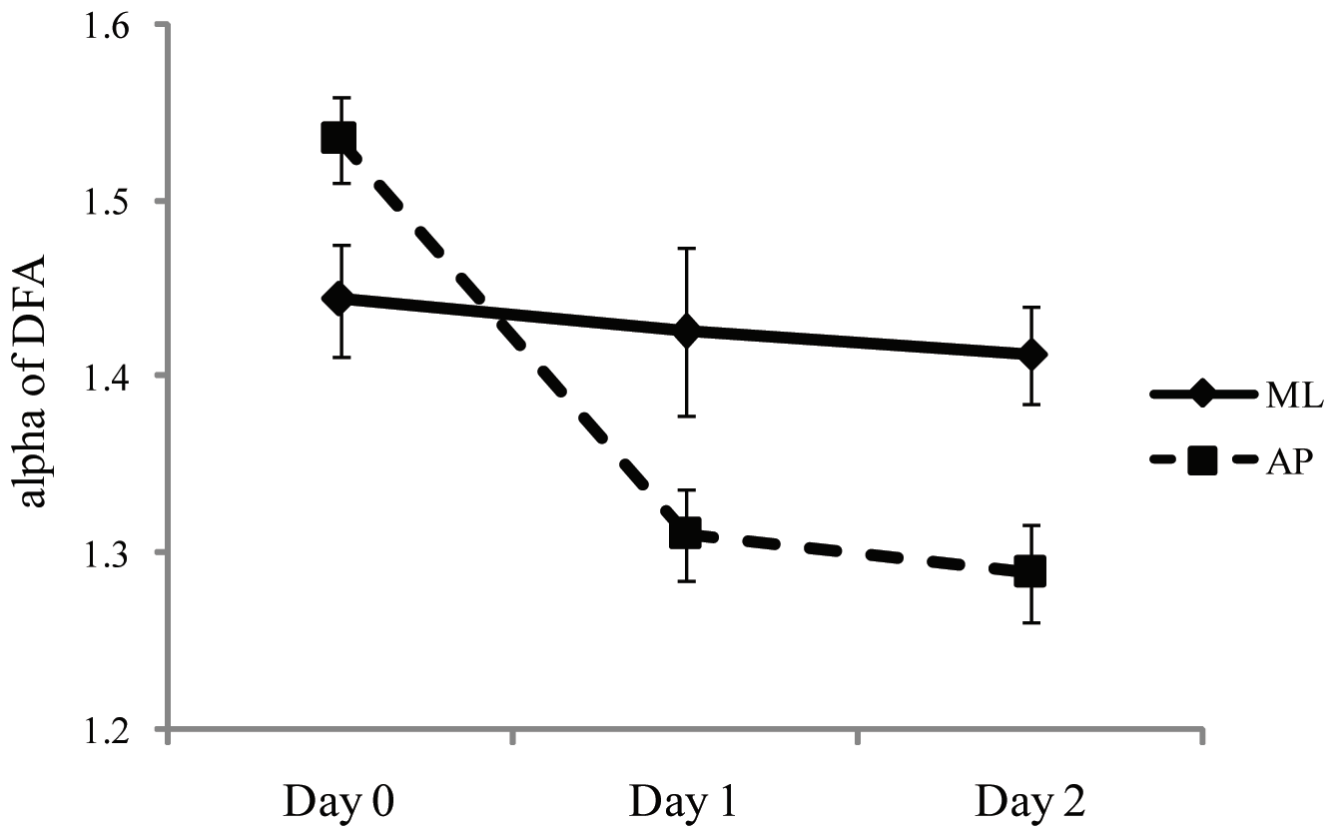
Experiment 2: Meanα of DFA for the AP and ML axes as a function of days. The figure illustrates the statistically significant interaction between axes and days. The error bars represent standard error of the mean.

For the temporal dynamics of sway, DFA revealed a significant main effect of days, F(2,56)  = 14.95, *p*<.001, partial η^2^  = 0.348. As shown in Figure 6, there was a reduction in self-similarity at sea, relative to values when the ship was at the dock. The main effect of axis was also significant, F(1,28)  = 11.15, *p*  = .002, partial η^2^  = 0.285, with mean α greater in ML (1.413) than in AP (1.372). Finally, the Days × Axis interaction was significant, F(2,56)  = 39.01, *p*<.001, partial η^2^  = 0.582. As can be seen in Figure 7, the relation between AP and ML that existed at the dock was reversed at sea. Post-hoc tests revealed that the transition from dock to sea had no effect on self-similarity in the ML axis. By contrast, in the AP axis, self-similarity at the dock (Day 0) was greater than on Day 1 or Day 2 (each *p*<.001), but self-similarity did not differ between the two days at sea (Day 1 vs. Day 2, *p* = .20).

## Discussion

We measured standing body sway in maritime novices before a voyage (when the ship was at the dock) and during each of the first two days at sea. At the dock, participants swayed more when viewing the horizon than when viewing a nearby target, consistent with terrestrial studies. At sea, overall sway was greater than when the ship was at the dock. In addition, sway when viewing the horizon was reduced, relative to sway during viewing of nearby targets. In this section, we discuss these findings in terms of processes of adaptation (i.e., the process by which people get their sea legs).

Before the voyage began (Day 0), the positional variability of the COP was comparable with that of terrestrial, laboratory-based research examining positional variability during viewing of nearby targets [29], [30], and with land-based testing of experienced maritime crewmembers [17]. In addition, positional variability was greater in the AP axis than in the ML axis, replicating a finding that is common in land-based studies [17], [41]. Finally, the temporal dynamics of sway were similar to those reported for young adults in terrestrial research [39]. These similarities confirm that, when tested on a stationary ship the body sway of our participants was typical.

### General effects of ship motion

The voyage brought about substantial changes in body sway. The most obvious effect of ship motion was an overall increase in the spatial magnitude of body sway, as reflected in an increase in the positional variability of the COP (Figure 3). The increase in spatial magnitude was paralleled by a decrease in the self-similarity of sway (Figure 6). These changes, caused by ship motion, resemble changes in body sway that are associated with healthy aging. On land, Lin et al. [39] evaluated body sway in young and elderly adults. As a group, elderly adults tended to sway more than young adults, as indicated by several measures of the spatial magnitude of sway. Lin et al. also evaluated the temporal dynamics of sway, using DFA, and found reduced self-similarity among elderly adults, relative to young adults. Elderly adults generally are regarded as have less stable control of body sway than younger adults. The results of Experiment 2 suggest that ship motion reduced the stability of body sway for young adults in ways similar to the effects of aging on body sway.

At sea (Days 1 and 2), the relative magnitude of sway in the AP and ML axes was reversed relative to body sway at the dock (Figure 5). A reversal was also observed in the temporal dynamics of sway (Figure 7): At the dock, self-similarity was greater in the AP axis than in the ML axis, while at sea the reverse was true. These effects may be related to the fact that in Experiment 2 participants were tested while facing the ship's stern. Chen and Stoffregen [16] compared body sway at sea when experienced mariners faced along the ship's long axis versus when they faced along the ship's short axis (athwartship). When facing with the ship's long axis (as in Experiment 2 of the present study) experienced mariners exhibited greater positional variability and greater self-similarity in the ML axis than in the AP axis. By contrast, when facing along the ship's short axis Chen and Stoffregen found that positional variability and self-similarity were greater in the AP axis than in the ML axis, as is commonly observed on land. In future research it will be interesting to determine whether maritime novices exhibit a similar type of orientation-specific response to ship motion.

### Effects of visual target distance

When the ship was at the dock (Day 0), participants exhibited greater positional variability of the COP when viewing the horizon, relative to sway when viewing the nearby target, consistent with the Grand Canyon effect. Thus, we successfully replicated effects of target distance on the magnitude of sway that have been observed on land among the general population [28], [29], [30] and when experienced mariners were tested on land [17].

At sea (Day 1 and Day 2), we observed a qualitatively different pattern: Participants exhibited reduced positional variability of the COP when viewing the horizon, relative to sway when viewing the nearby target (Figure 4). In achieving this change novice mariners reversed a lifetime of experience in relations between postural control and visual information. A similar effect was observed when experienced mariners were tested at sea [17].

At sea, relations between body sway and the distance of visual targets did not change across days. Thus, the qualitative change from the land-based pattern to the sea-based pattern appeared to have occurred (and to be complete) within the first 24 hours of the voyage. We conclude that in less than 24 hours people learned to use the horizon to control body sway. In future research it will be important to identify and study the process of transition from the land-based pattern to the sea-based pattern. After the beginning of a voyage, what is the time course of the process by which the horizon comes to be used in the control of body sway? Is this change gradual or sudden? Answers to these questions will require that measurements of body sway be taking more often, such as repeated testing on an hourly basis.

There were no significant effects of the distance of visual targets on the temporal dynamics of sway, either at the dock or at sea. Thus, the horizon affected the spatial magnitude of sway, but not its temporal dynamics. Studies on land have sometimes found that variations in visual tasks can influence the temporal dynamics of sway [32], [33]; however, those studies did not include variations in the distance of visual targets. Future research is needed to understand why the temporal dynamics of body sway are affected by some parameters of visual tasks but not by others.

Experiment 2 revealed that, on going to sea, maritime novices increased the spatial magnitude of their body sway (Figure 3) and (in the AP axis) decreased the self-similarity of COP positions (Figure 7). While at sea, looking at the horizon was associated with reduced spatial magnitude of body sway, consistent with the behavior of experienced mariners [17]. These effects were established within 24 hours of the beginning of the voyage and, thereafter, did not change over time.

## Experiment 3

Medicine and science have freed us from countless maladies, but seasickness remains. Seasickness can occur at any point in a voyage, even among seasoned mariners [19], but it is most closely associated with the beginning of voyages, that is, with the period during which people are getting their sea legs. Data on the incidence and phenomenology of seasickness are widely available [19], [42]. For this reason, in Experiment 3 we did not focus on these aspects of seasickness; rather, we attempted to understand relations between seasickness and body sway.

### Motion sickness and body sway

Motion sickness is widely associated with unstable control of the body. Many studies have documented changes in body sway following exposure to nauseogenic motion stimuli. For example, virtual environments sometimes give rise to motion sickness, and exposure to virtual environments tends to increase body sway (for a review, see [43]). The fact that unstable control of the body follows the onset of motion sickness is not surprising to anyone who has suffered from the malady, and has not been thought to have significance for theories of motion sickness etiology: Unstable sway that follows motion sickness cannot be the cause of motion sickness. Greater theoretical significance accrues to the fact that unstable control of body sway can precede motion sickness.

On land, motion sickness can be preceded by unstable body sway. Owen, Leadbetter, and Yardley [44] used questionnaires to assess participants' generalized motion sickness susceptibility. Numerical ratings of motion sickness susceptibility derived from the questionnaires were positively correlated with the magnitude of body sway. Similarly, Yokota, Aoki, Mizuta, Ito, and Isu [45] used questionnaire data to classify participants into high- and low-susceptibility groups. These groups differed in postural responses to oscillatory visual motion stimuli. Owen et al. [44] and Yokota et al. [45] did not attempt to induce motion sickness in the laboratory. In other studies, researchers have measured unperturbed body sway before participants were exposed to visual motion stimuli that induced motion sickness in some participants [46], [47], [48]. Pre-exposure body sway differed between participants who (later) became motion sick and those who did not. These studies, together with those of Owen et al., [44] and Yokota et al. [45] suggest that there may be generalized differences in body sway between individuals who are susceptible to motion sickness and those who are not. All of these studies were conducted in the laboratory; none specifically addressed relations between body sway and seasickness.

Tal, Bar, Nachum, Gil, and Shupak [49] evaluated standing body sway in naval recruits at the beginning of their training, and compared these data to subsequent reports of seasickness during training cruises. They found no relation between pre-voyage sway and subsequent seasickness. On the basis of their findings Tal et al. [49] argued that seasickness cannot be predicted from land-based data on body sway. However, their analysis of body sway was limited to measures of the spatial magnitude of sway, and to stance during moving platform posturography. Before accepting their conclusion we felt it was appropriate to consider sway in other situations, and measures of the temporal dynamics of sway as well as its spatial magnitude.

It is important to note that the stability of postural activity can be evaluated in many ways [39], [50]. Some of these are related to the spatial magnitude of movement, while others are related to the temporal dynamics of movement. The magnitude and dynamics of movement are equally real but qualitatively different, such that one cannot be reduced to the other. Several authors have suggested that the temporal dynamics of movement may be related to a variety of pathological conditions [51], [52], including motion sickness [53], [54]. Before exposure to potentially nauseogenic motion stimuli we have identified differences in the temporal dynamics of body sway related to the subsequent incidence of motion sickness. Stoffregen et al. [47] and Villard et al. [48] found greater self-similarity in body sway among participants who later became motion sick than among those who did not.

### Seasickness and the horizon

As noted earlier, maritime novices often are advised to keep the horizon in view as a means to avoid seasickness [19], [26], [27], [55]. This advice and the underlying anecdotal reports suggest that increased stability in control of the body may help to prevent seasickness or, conversely, that unstable control of the body may increase the risk of seasickness. In ship simulators the ability to see the stable surroundings of the motion platform can reduce motion sickness [56], while in experienced mariners [17] and maritime novices (Experiment 2 of the present study) body sway was reduced during stance on the open deck of a ship when looking at the horizon. Taken together, these findings suggest that susceptibility to seasickness may be related to individual differences in postural responses to the visible horizon. In Experiment 3, we evaluated this hypothesis by including target distance (nearby target vs. the horizon) as an independent variable in our analysis of relations between seasickness and body sway.

### Seasickness and stance width

In the laboratory, wider stance width is associated with a reduced incidence of visually induced motion sickness. Stoffregen et al. [47] assigned participants to different stance width groups (5 cm, 17 cm, or 30 cm) during exposure to potentially nauseogenic visual motion (oscillation of the visible surroundings in a moving room). The percentage of participants reporting motion sickness was lower with wider stance, and higher with narrow stance width. This effect raises the possibility that persons who choose wider stance might have a reduced susceptibility to seasickness. In Experiment 3 we evaluated this hypothesis.

### Summary

We sought to relate the severity of seasickness to variations in stance width and body sway. We did this in two qualitatively different ways. First, we evaluated the hypothesis that seasickness would be related to stance width or body sway prior to the beginning of the voyage (i.e., before exposure to ship motion). To evaluate this hypothesis, we used the severity of seasickness on Day 1 as an independent variable in analyses of stance width and body sway from Day 0. Second, we evaluated the hypothesis that seasickness would be related to stance width or body sway at sea (i.e., after the onset of seasickness). To evaluate this hypothesis, we used the severity of seasickness on Day 1 and Day 2 as an independent variable in analyses of stance width and body sway from Day 1 and Day 2.

The ship departed Nassau in the evening. In part for this reason, we were not able to collect data on body sway at sea before the onset of seasickness. Thus, we were not able to evaluate the hypothesis that body sway at sea (measured before anyone became ill), would be related to the severity of subsequent seasickness.

There are many measures of the severity of seasickness symptoms, but there are no widely accepted metrics for seasickness incidence [57]. In part this is because ship motion is continuous from the moment of departure; it can vary but rarely disappears entirely. Thus, a simple yes/no dichotomy or sick/well categorization is less credible than in laboratory research. To accommodate the characteristics of seasickness we assigned participants to different groups based on the overall severity of seasickness.

## Method

### Participants

We analyzed data from a total of 33 participants (nine males and 24 females); with mean age 20.61 years (SD  = 1.95 years) and mean height 168.80 m (SD  = 8.13 cm). The number of participants whose data were included in each analysis is reported below.

### Procedure

We used a seasickness questionnaire to collect data on motion sickness symptomology. On the questionnaire, participants were asked to indicate their overall experience with seasickness over the previous 24 hours, choosing from among four options: None at all, mild, moderate, or severe. We used these ratings to assign participants to seasickness groups, as described below. Participants also rated the severity of 14 individual symptoms that are associated with motion sickness. These symptoms were a subset of questions from the Simulator Sickness Questionnaire [58]. Participants completed the seasickness questionnaire prior to postural testing on Day 0, Day 1, and Day 2.

### Data analysis

We conducted new analyses of stance width data from Experiment 1, and of body sway data from Experiment 2. In these analyses we treated seasickness severity as an independent variable. In Experiment 3 we report only main effects and interactions that included this variable. We conducted separate analyses for each day of postural testing, that is, we did not include days as an independent variable. To determine whether seasickness was preceded by differences in stance width or body sway, we analyzed data from Day 0, classifying the data into groups based on reports of seasickness from Day 1. To determine whether the experience of seasickness influenced postural activity at sea, we analyzed data on stance width and body sway from Day 1 in relation to seasickness on Day 1, and we analyzed data on stance width and body sway from Day 2 in relation to seasickness on Day 2.

## Results

### Subjective reports

To achieve comparable sample sizes, on each day we clustered participants into three groups based on their overall level of seasickness; None, Mild, and Moderate/Severe. At the dock (Day 0) each participant indicated that their level of seasickness was None. At sea, on Day 1 eight participants (24%) reported their level of symptoms as None. Thirteen participants reported mild symptoms, while 12 participants reported moderate or severe motion sickness, so that the overall incidence of seasickness (i.e., any vs. none) was 25 out of 33 (76%). On Day 2 eight participants (26%) indicated that their level of symptoms was None. Fifteen reported mild motion sickness, while seven indicated that they had moderate or severe motion sickness, so that the overall incidence of seasickness was 22 out of 30 (73%). Data on motion history for the three groups are reported in Table 1.

**Table 1:** Motion sickness history.

Seasickness severity group (Day 1 at sea)	Experience at sea	Seasickness history	General susceptibility to motion sickness
None (N = 8)	1.75±0.71	4.63±0.74	4.5±0.53
Mild (N = 13)	2.31±1.03	4.17±0.83	3.92±0.76
Moderate/Severe (N = 12)	2.75±1.06	3.00±1.08	3.04±1.10

Experience at sea was rated on a 4-point scale, where 4 =  no previous experience. Seasickness history was rated on a 5-point scale, where 5 =  I have never been motion sick. General susceptibility to motion sickness was rated on a 5-point scale where 1 =  maximum susceptibility.

**Figure 8:**
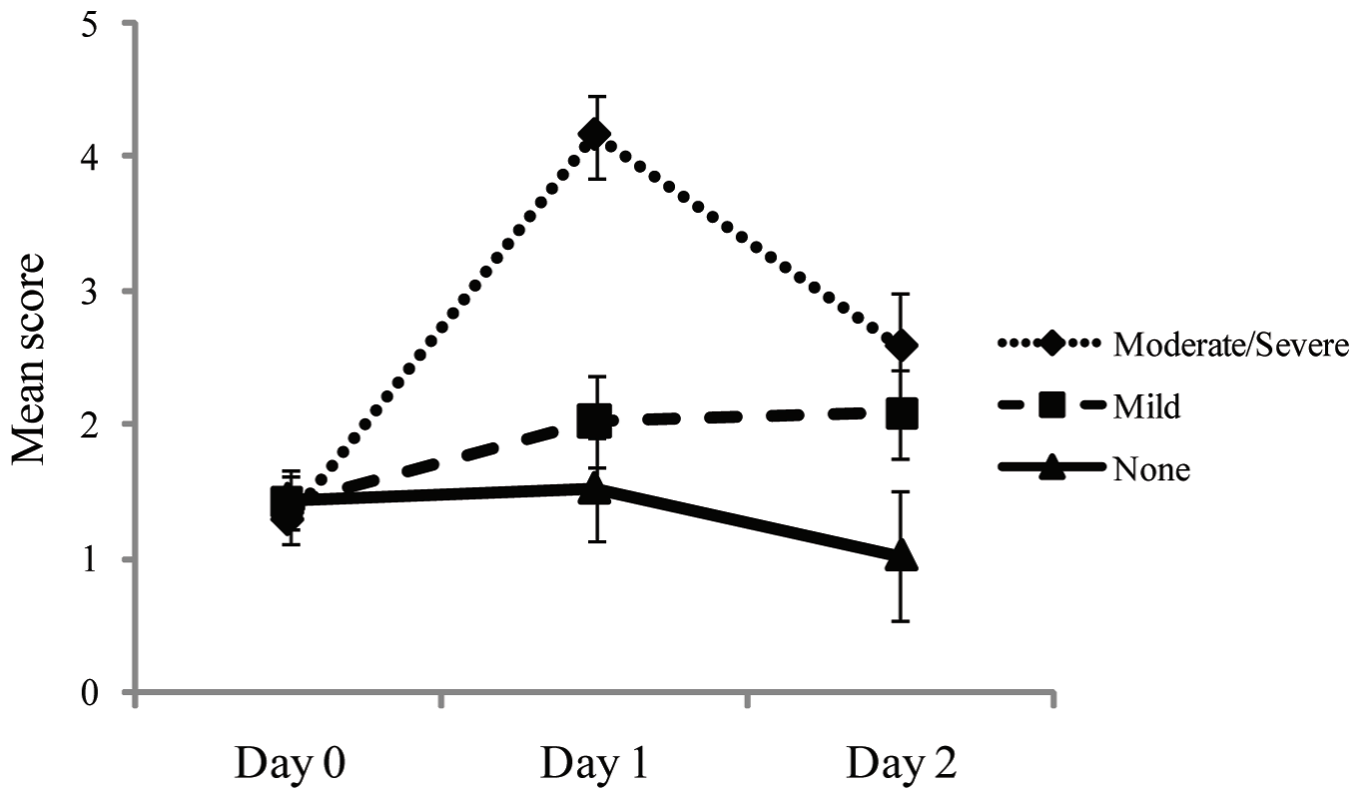
Experiment 3: Mean symptom ratings for the three seasickness severity groups as a function of days. The error bars represent standard error of the mean.

Ratings of the severity of individual symptoms are summarized in Figure 8. For each participant on each day, we computed the mean score across the 14 questions. Combining across seasickness severity groups, we used the Wilcoxon signed ranks test to evaluate changes in symptom severity across days. Symptoms were more severe on Day 1 than on Day 0, *z* = 4.10, *p*<.001. Symptom severity declined on Day 2, relative to Day 1, *z* = 3.36, *p* = .001. Day 0 and Day 2 did not differ, *z* = 1.20, *p* = .231. We used the Kruskal-Wallis test to evaluate differences in symptom severity between the three seasickness severity groups. On Day 0, symptom severity did not differ between the three seasickness severity groups, χ^2^ = 1.71, *p* = .425. On Day 1, symptoms differed between groups, χ^2^ = 19.40, *p*<.001. On Day 2, symptoms again differed between groups, χ^2^ = 14.15, *p* = .001.

### Seasickness and stance width

For stance width we conducted one-factor ANOVAs on seasickness groups.

#### Day 0 stance width in relation to Day 1 seasickness

There were 33 participants. There were no significant effects.

#### Day 1 stance width in relation to Day 1 seasickness

**Figure 9:**
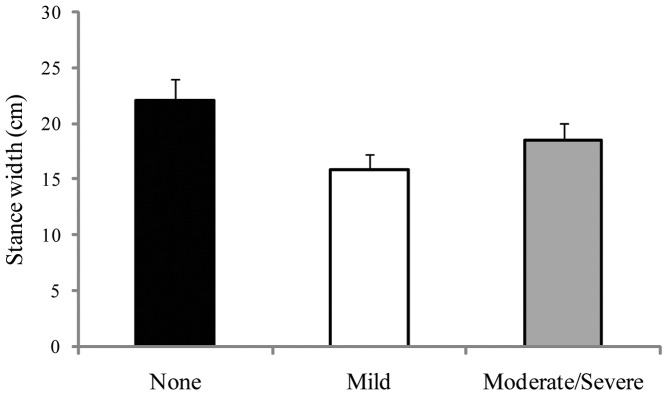
Experiment 3: Mean stance width (distance between the midlines of the heels) on Day 1 at sea, as a function of seasickness severity groups. The figure illustrates the statistically significant effect of seasickness severity groups. The error bars represent standard error of the mean.

There were 33 participants. We found a significant main effect of Seasickness Group, *F*(1,2)  = 3.36, *p* = .048 (Figure 9). Multiple comparisons revealed that stance width for the None group was greater than for the Mild group, *p* = .016. The Mild group did not differ from the Mod/Severe group, *p* = .186, and the None group did not differ from the Mod/Severe group, *p* = .212.

#### Day 2 stance width in relation to Day 2 seasickness

There were 31 participants. There were no significant effects.

### Seasickness and body sway

For body sway we conducted three-factor ANOVAs with factors target distance (near target vs. horizon), axis (AP vs. ML), and seasickness group (none, mild, moderate/severe).

#### Day 0 body sway in relation to Day 1 seasickness

**Figure 10:**
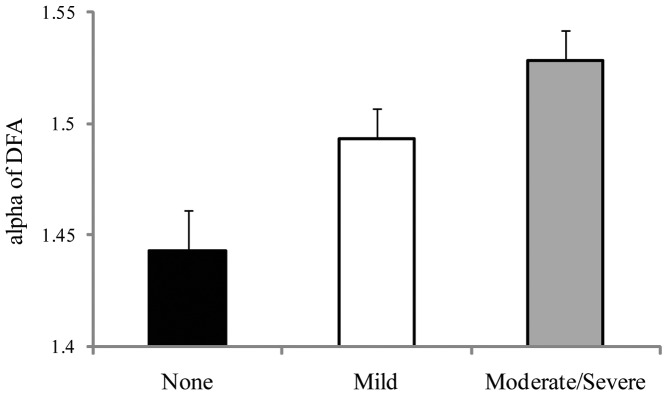
Experiment 3: Meanα of DFA on Day 0 (before the voyage began) for the three seasickness severity groups. The figure illustrates the statistically significant effect of seasickness severity groups. The error bars represent standard error of the mean.

**Figure 11:**
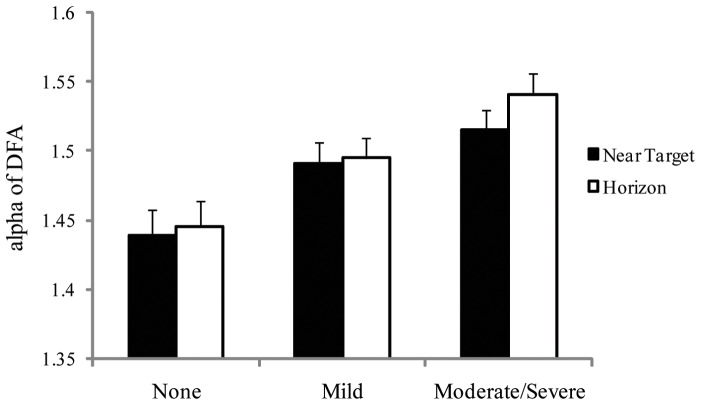
Experiment 3: Meanα of DFA on Day 0 (before the voyage began) during viewing of the nearby target and the horizon, for the three seasickness severity groups. The figure illustrates the statistically significant interaction between seasickness severity groups and visual targets (near target vs. horizon). The error bars represent standard error of the mean.

There were 33 participants. For positional variability of the COP, there were no significant effects. For the temporal dynamics of the COP, DFA revealed a significant main effect of seasickness severity groups, F(2,31)  = 6.98, *p* = .003, partial η^2^  = 0.317, which is illustrated in Figure 10. Post-hoc tests (multiple comparisons) revealed that the None group differed from the Mild group, *p* = .035, and from the Moderate/Severe group, *p*<.001. The Mild and Moderate/Severe groups did not differ from each other, *p* = .086. In addition, the Seasickness Group × Target Distance interaction was significant, F(2,31)  = 3.03, *p* = .045, partial η^2^  = 0.168 (Figure 11). Post-hoc tests revealed that α did not differ between the near target and horizon conditions for the None group or for the Mild group. For the Mod/Severe group, the mean difference was significant, *p* = 0.015; for this group, α was greater when looking at the horizon than when looking at the nearby target.

#### Body sway at sea in relation to seasickness

There were 31 participants. We found no significant effects relating body sway on Day 1 to the severity of seasickness symptoms on Day 1. Similarly, we found no significant effects relating body sway on Day 2 to the severity of seasickness symptoms on Day 2.

## Discussion

We classified participants into three groups based on the overall severity of seasickness experienced during the voyage. Before the voyage began (Day 0), these differences in seasickness severity were preceded by differences in the temporal dynamics of body sway. Also on Day 0, among participants who subsequently reported moderate or severe seasickness the temporal dynamics of body sway were influenced by the distance of visual targets (nearby target vs. the horizon). Finally, on the first day at sea (Day 1) stance width was narrower among participants who reported mild seasickness than among those who reported no seasickness.

### Seasickness incidence

If we equate the incidence of seasickness with the presence of any level of seasickness symptoms, then the incidence of seasickness in Experiment 3 was comparable with previous studies on ships at sea [19]. For example, on a 3000-ton vessel in mild seas (sea states 2 and 3 on the Beaufort scale [22]), Attias, Gordon, Ribak, Binah, and Rolnick [59] found that 53% of those not receiving seasickness medication were sick on the first two days at sea.

### Stance width and seasickness

Prior to the beginning of the voyage (Day 0) there were no effects relating stance width to subsequent seasickness. By contrast, on the first day at sea participants who reported no seasickness (the None group) had greater stance width than participants in the Mild seasickness group (Figure 9). This effect was short-lived: By Day 2 there was no longer a significant difference in stance width between the seasickness groups. We assessed stance width and seasickness in the same session. For this reason it was not possible to determine causality in the significant relation between stance width and seasickness on Day 1. Future research should address this issue directly, using very early measures of self-selected stance width, or experimenter controlled between-participants variations in stance width.

### Body sway and seasickness

We measured the self-similarity of COP positions when the ship was at the dock (Day 0). We compared these pre-voyage data on body sway with participants' reports of seasickness on each of the first two days at sea. We found that the self-similarity of pre-voyage body sway differed as a function of Day 1 membership in the three seasickness severity groups. Post-hoc tests revealed that self-similarity was lower among participants who did not experience seasickness (the None group), and higher among participants in either the Mild or the Moderate/Severe groups. That is, self-similarity differed between participants with any level of seasickness and those with no seasickness, and this difference existed before the beginning of the voyage, that is, before participants were exposed to ship motion. We discuss the theoretical significance of this effect in a later section.

In contrast to our analysis of the self-similarity of body sway, we found no evidence that pre-voyage data on positional variability were related to the subsequent experience of seasickness. The absence of an effect relating seasickness to positional variability contrasts with studies that have identified pre-exposure differences between susceptible and insusceptible individuals in measures of sway magnitude [44], [45], [46], [47], [48], [60]. Unlike these previous studies, Experiment 3 focused on seasickness and did not include any other type of motion sickness. The differing patterns of results relating to the spatial magnitude of body sway may be related to this difference in study design.

Tal et al. [49] analyzed the spatial magnitude of body sway before a sea voyage and found no differences between participants who subsequently reported seasickness and those who did not. Tal et al. argued that seasickness cannot be predicted from postural data collected on land prior to a voyage. With respect to the magnitude of body sway our results are compatible with their conclusion. However, with respect to the temporal dynamics of body sway our results support a different conclusion. Body sway is complex, and can be described using a wide variety of dependent variables. Some measures (e.g., the velocity and positional variability of the COP), are correlated but others (e.g., positional variability and temporal self-similarity of the COP) differ qualitatively. This fact raises questions about how we define stability and instability in the context of body sway [52], [53].

Before departure (Day 0), our analysis of the temporal dynamics of body sway revealed that values of α for the None group were representative of young adults on land [39]. Values of α for the Mild group and the Mod/Severe group were abnormally high. Similar effects have been observed in the context of visually induced motion sickness [47], [48]; the results of Experiment 3 extend these effects to the domain of seasickness. These effects are compatible with the idea that individuals susceptible to motion sickness have more rigid or deterministic control of body sway.

### Influence of the horizon

In Experiment 2, we found no effects of visual target distance on the temporal dynamics of body sway, either before or during the voyage. By contrast, Experiment 3 revealed that the temporal dynamics of pre-voyage sway were influenced by the horizon, but only among participants who subsequently experienced more severe seasickness (Figure 11). For these participants, the self-similarity of body sway on Day 0 was greater when viewing the horizon than when viewing the nearby target. This effect provides the first experimental evidence of a link between seasickness, body sway, and the visible horizon. Given the results of Experiment 2 it is perhaps surprising that this link was observed only in the temporal dynamics of body sway, and only in relation to body sway before the voyage began. These complex relations can be addressed only through additional research.

### Motion sickness etiology

Seasickness is a form of motion sickness, and so understanding of the precursors of seasickness may help to inform general theories of motion sickness etiology. Like other forms of motion sickness seasickness typically has been interpreted in terms of the concept of intersensory conflict. In the sensory conflict theory of motion sickness, it is argued that behavior in normal environments gives rise to a set of internal expectations (often referred to as an internal model or neural store; e.g., [61] about relations between stimulation of different perceptual systems (e.g., visual, vestibular, somatosensory). The theory claims that in moving environments (e.g., on a ship) these expectations are violated, that is, the pattern of intersensory stimulation experienced in moving environments is believed to conflict with the pattern expected on the basis of past experience [62]. The magnitude of this hypothetical conflict is believed to scale to the incidence and severity of consequent motion sickness [61]. Despite its intuitive appeal, models based on sensory conflict have low predictive validity [63], and interventions inspired by the theory (intended to reduce motion sickness incidence and/or severity) have had limited success [64]. Of special relevance to the present study, the sensory conflict theory of motion sickness does not motivate the hypothesis that variations in the control of posture may precede the subjective symptoms of motion sickness.

Overall, the results of Experiment 3 are consistent with the postural instability theory of motion sickness [53], which predicts that unstable control of bodily orientation should precede the onset of subjective symptoms of motion sickness. Our results do not establish a causal link between body sway and seasickness but they do pose challenges for any theory of motion sickness etiology.

## Experiment 4

Persons who are adapted to ship motion often find that they experience a period of re-adaptation on returning to land. This re-adaptation, known as *mal de debarquement*, comprises a variety of phenomena. These include subjective experiences, such as the feeling that the land is moving underneath [65] and objective effects, such as changes in postural control [66]. Gordon, Spitzer, Doweck, Melamud, and Shupak [67] found that 72% of maritime crewmembers reported experiencing some level of mal de debarquement.

In Experiment 4, our primary purpose was to assess relations between body sway and mal de debarquement. Nachum et al. [66] evaluated standing posture before and after a sea voyage, comparing participants who were susceptible and those who were insusceptible to mal de debarquement. Postural testing consisted of moving platform posturography using a protocol known as the sensory organization test, or *SOT*. Before a voyage, the postural sway of susceptible and insusceptible groups differed when participants stood with eyes closed on a platform that rotated about the ankle joint axis in proportion to the participant's spontaneous body sway in the AP axis (condition 6 of the SOT). Nachum et al. [66] noted that this result was compatible with the postural instability theory of motion sickness but also suggested that it might indicate long-term aftereffects of previous sea voyages.

Experiment 4 differed from the study of Nachum et al. [66] in terms of the test conditions, and in terms of the dependent variables that we used to evaluate body sway. Nachum et al. [66] measured postural activity only on land, whereas we measured body sway both before and during a voyage. Nachum et al. [66] analyzed postural activity exclusively in terms of the spatial magnitude of sway (measured sway as a proportion of the maximum sway possible during feet together stance). We evaluated the spatial magnitude of sway (operationalized as the positional variability of the COP), but in addition we analyzed the temporal dynamics of sway, using DFA. Finally, while Nachum et al. [66] limited their analysis to body sway in the body's AP axis we analyzed activity in both the AP and ML axes.

Mal de debarquement is widely understood to be a form of motion sickness [54], [62], [65], [66], [67]. Thus, the postural instability theory of motion sickness predicts that postural activity should differ between persons susceptible to mal de debarquement and those who are not, and that differences should exist before the onset of subjective symptoms of mal de debarquement. In experiment 4 we evaluated this prediction separately with regard to postural activity before the beginning of the voyage, and during the voyage.

## Method

### Participants

Four males and 20 females from Experiment 2 completed and returned the mal de debarquement questionnaire, and so were included in Experiment 4. For these 24 participants the mean age was 20.54 years (SD  = 2.25 years), and the mean height was 168.28 m (SD  = 7.93 cm).

### Procedure

The ship arrived at Dominica at 08∶00 and disembarkation began immediately, with most students having disembarked by 10∶00. Mal de debarquement questionnaires were delivered to participants' cabins during the afternoon, and were completed and returned before 08∶00 the following day.

To assess mal de debarquement we used a questionnaire similar to that developed by Gordon et al. [65]. Questions addressed the specific symptoms experienced, the number of times symptoms were experienced, and the total duration of symptoms. Following Nachum et al. [66], participants who reported experiencing mal de debarquement symptoms for at least 120 minutes during the first day ashore were assigned to the High-MD group. The Low-MD group comprised participants who reported experiencing mal de debarquement symptoms for 30 minutes or less during the first day ashore.

## Results

### Subjective reports

Nineteen participants (79%) reported experiencing mal de debarquement symptoms for 30 minutes or less, and were assigned to the Low-MD group. Five participants (21%) reported experiencing mal de debarquement symptoms for 120 minutes or more, and were assigned to the High-MD group. There were no intermediate values, that is, none of the participants reported experiencing symptoms for more that 30 minutes but less than 120 minutes. A similar bimodal distribution was reported by Gordon et al. [67].

The simple correlation between the duration of MD symptoms (in minutes) and Day 1 seasickness severity ratings (None, Mild, Moderate, Severe), *r*  = .518, was significant, *p* = .009. This relation confirmed previous reports that persons at risk for seasickness are also at risk for mal de debarquement [65], [66], [67].

### Body sway in relation to mal de debarquement

#### Day 0 body sway in relation to mal de debarquement

Separately for positional variability and DFA, we conducted 3-way ANOVAs on target distance (near target vs. the horizon), axis (AP vs. ML), and group (High-MD vs. Low-MD). We found no significant effects relating to the mal de debarquement groups for positional variability, or for DFA.

#### Days 1–2 body sway in relation to mal de debarquement

Experiment 2 revealed no significant differences in body sway between the two days at sea (Day 1 and Day 2). For this reason, in evaluating relations between mal de debarquement and body sway at sea, we collapsed across days at sea. Separately for positional variability and DFA, we conducted 3-way ANOVAs on target distance (near target vs. the horizon), axis (AP vs. ML), and group (High-MD vs. Low-MD).

**Figure 12:**
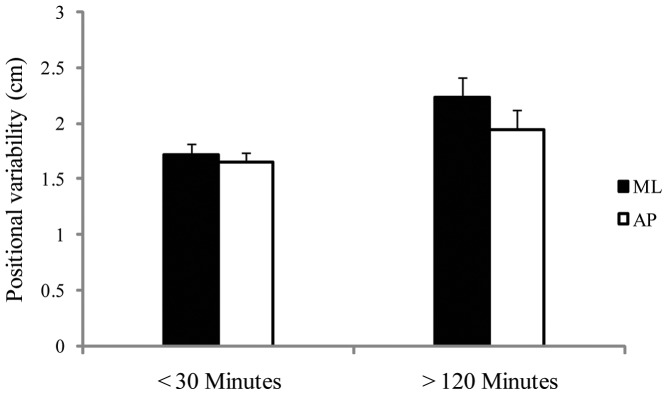
Experiment 4: Mean positional variability in the AP and ML axes for participants who experienced mal de debarquement for less than 30 minutes (the Low-MD group) or more than 120 minutes (the High-MD group). The figure illustrates the statistically significant main effect of groups (<30 minutes vs. >120 minutes), and the statistically significant interaction between groups and body axes (AP vs. ML). The error bars represent standard error of the mean.

For positional variability, we found a significant difference between the mal de debarquement groups, F(1,22)  = 4.37, *p*  = .048, partial η^2^  = 0.152. Positional variability for the High-MD group (mean  = 2.09 cm, SD  = 0.173) was greater than for the Low-MD group (mean  = 1.70 cm, SD  = 0.089). In addition, we found a significant Group × Axis interaction, F(1,22)  = 6.39, *p* = .019, partial η^2^  = 0.225 (Figure 12). For the High-MD group, the difference between AP and ML was larger than for the Low-MD group. The High-MD group exhibited increased sway in the ML axis and reduced sway in the AP axis, while for the Low-MD group the positional variability of body sway in the AP and ML axes tended to be equal.

For the temporal dynamics of sway, DFA revealed a main effect of Groups, F(1,22)  = 5.77, *p* = .025, partial η^2^  = 0.208. Body sway for the High-MD group (mean α  = 1.34, SD  = 0.014) was less self-similar than for the Low-MD group (mean α  = 1.41, SD  = 0.027). There were no other significant effects.

## Discussion

After disembarking from the ship, participants reported their experience with subjective symptoms of mal de debarquement. We used these subjective reports to evaluate measures of body sway collected before the beginning of the voyage (Day 0), and during the voyage (Days 1–2). Sway on Day 0 did not differ between the Low-MD and High-MD groups. By contrast, body sway at sea (Days 1–2) differed between the two mal de debarquement groups.

### Mal de debarquement in relation to sway before the voyage

At the dock (Day 0) body sway did not differ as a function of participants' subsequent experience of mal de debarquement. Nachum et al. [66] also did not find differences in body sway between groups during stance on a stationary surface (i.e., SOT conditions in which the posturographic platform was stationary).

### Mal de debarquement in relation to sway during the voyage

During the voyage, sway differed between participants as a function of their subsequent level of mal de debarquement symptoms. At sea, greater positional variability in sway was associated with greater duration of mal de debarquement symptoms. In addition, the group × axis interaction revealed that the difference in positional variability between the AP and ML axes was greater for participants with longer duration mal de debarquement symptoms (Figure 12). This interaction indicates that mal de debarquement was not related solely to the spatial magnitude of body sway.

At sea, the temporal dynamics of body sway (α of DFA) differed between the High-MD and Low-MD groups. This finding confirms that mal de debarquement was not exclusively related to the spatial magnitude of body sway. The effect is remarkable also because of its direction: The self-similarity of body sway was negatively related to the duration of mal de debarquement. This pattern is in sharp contrast to our results with seasickness: In Experiment 3, the self-similarity of body sway (on Day 0) was positively related to the severity of seasickness. This qualitative difference in the direction of effects relating body sway to seasickness and mal de debarquement underscores the powerful effects of ship motion on control of the body. The difference is the more remarkable given that we found a significant correlation between the severity of seasickness and the duration of mal de debarquement symptoms: Divergent relations between body sway, seasickness, and mal de debarquement occurred in the same individuals.

Our effects relating mal de debarquement to body sway resemble those reported by Nachum et al. [66], in the sense that in both studies relations between sway and mal de debarquement were observed during stance on moving surfaces; a sway-referenced force platform in the study of Nachum et al., and a ship at sea in the present study. This similarity suggests that susceptibility to mal de debarquement may be related to individual differences in perceptual-motor adaptation to vehicle motion. This possibility is consistent with the broader hypothesis that susceptibility to different forms of motion sickness may be related to situation-specific individual differences in the capacity for perceptual-motor adaptation and learning [54].

### General Discussion

We conducted the first experimental study of the processes by which maritime novices get their sea legs. In a within-participants design we examined changes in body sway and in positioning of the feet associated with the beginning of a sea voyage, and we related these data to reports of seasickness and mal de debarquement. Using this integrated approach we identified several novel effects. At sea, novice mariners rapidly adopted a wider stance. With equal rapidity, ship motion brought about a qualitative change in the influence of the visible horizon on body sway. Our results revealed that body sway before the beginning of the voyage was related to the severity of subsequent seasickness. In addition, we found the first experimental evidence that seasickness may be related to effects of the visible horizon on body sway. Finally, we found that the spatial magnitude and temporal dynamics of body sway at sea differed as a function of the duration of subsequent mal de debarquement. In this section we discuss relations between the experiments, and relations between the experiments and more general issues, both basic and applied.

### The visible horizon as a referent for perception and control

Prior to the beginning of a voyage participants exhibited greater body sway when looking at the horizon than when looking at a nearby target, replicating the terrestrial Grand Canyon effect [17], [31]. At sea, sway was greater during viewing of the nearby target, and was reduced during viewing of the horizon: The Grand Canyon effect was reversed. These results undermine the hypothesis that the terrestrial Grand Canyon effect is related to the detectability of the optical consequences of body sway in relation to the horizon [31]. The results also raise questions about the referents that we use for the perception and control of body orientation, in general.

Why is the visible horizon useful in the control of body sway at sea? Vehicle motion creates inertial force, which affects bodily orientation. This effect might be interpreted as a local factor that applies only to vehicular travel. However, in terms of physical constraints on orientation the effect reflects a more general phenomenon. Our analysis of the physical constraints that govern dynamic orientation relative to the direction of balance differs from classical analyses, which focus on static orientation relative to the direction of gravity [5]. For detailed discussions, see [7], [8], [68].

In general (i.e., both on vehicles and on the surface of the Earth), dynamic orientation is constrained by the gravitoinertial force vector (the vector sum of gravitational and inertial forces), which determines the direction of balance. Typically, postural control actions serve to maintain the body in alignment with the direction of balance, and the subjective experience of bodily orientation is more closely related to this alignment than to alignment relative to the direction of gravity [20]. When standing on the surface of the Earth, changes in orientation arise from body movement. On vehicles (including ships), the surface of support is also in motion and, therefore, changes in orientation arise (simultaneously) both from body movement and from motion of the vehicle. Control of body posture is simplified if we can differentiate body sway from motion of the support surface [68]. At sea, a visible horizon may make it easier to differentiate body sway (relative to the ship) from motion of the ship (relative to the Earth), which in turn would make it easier to maintain the body in alignment with the direction of balance [7].

Presumably, our novice participants had spent their lives modulating sway on the basis of target distance (where *target*  =  any object of regard). Despite this lifetime of experience participants accomplished a qualitative change in relations between target distance and body sway, and did so within the first 24 hours of exposure to ship motion. It may be that rapid adaptation to sea travel was potentiated, at least in part, by prior experience with travel in other vehicles, such as automobiles. In automobiles, as on ships, the body must be stabilized relative to the vehicle [68], and a visible horizon may facilitate this control. It is important to note, however, that there are several important differences between automobiles and ships. For example, the motion characteristics of automobiles and ships, while overlapping, are not identical. Ships and automobiles differ also in the duration of exposure: Continuous exposure over several days is ordinary for sea travel, but unheard of in automobiles. Finally, sea travel typically includes the control of body posture in multiple configurations (standing, sitting, lying down), whereas automobiles typically are associated exclusively with sitting. These differences suggest that any carryover in adaptation from automobiles to sea travel must exist at a very abstract level. It would be interesting, using within-participants designs, to compare the control of seated body sway relative to the visible horizon in automobiles and on ships.

The qualitative reversal of the terrestrial Grand Canyon effect that we observed at sea can motivate new research on postural control in vehicles. Is the horizon used to stabilize standing body sway in terrestrial vehicles, such as buses and trains, and in aerial vehicles, such as aircraft? Terrestrial and aerial vehicles typically entail sitting rather than standing, and it would be useful to know whether the visible horizon can be used for the control of seated posture in vehicles; similar research might be conducted with seated participants on ships.

### Body sway and seasickness

Seasickness was preceded by distinctive patterns of body sway. Before exposure to ship motion, the temporal dynamics of body sway were related to the severity of subsequent seasickness (Figure 10). The self-similarity of sway was greater among participants whose seasickness was more severe. This result is consistent with terrestrial studies relating body sway to visually induced motion sickness [47], [48], [60], and with studies relating body sway to generalized motion sickness susceptibility [44], [45].

We generated the first empirical evidence of a relation between seasickness, body sway, and the visible horizon (Figure 11). For participants who experienced little or no seasickness, the temporal dynamics of body sway on land did not differ when looking at the horizon versus a nearby target. Among participants who later experienced more severe seasickness the self-similarity of sway was greater when looking at the horizon (on land) than when looking at the nearby target.

Our results raise broader questions about how motion sickness may be related to the visible horizon in different situations. Looking at the horizon is recommended as a preventative measure for seasickness, but also as a preventative measure for motion sickness in terrestrial vehicles [69], [70]. Our results motivate new research relating the visible horizon to body sway and motion sickness in automobiles and aircraft. Such questions cannot easily be answered in the context of simulators and virtual environments, in part because these technologies tend to elicit motion sickness in situations that are not associated with motion sickness in the corresponding physical world.

The effects observed in Experiment 3, together with similar effects obtained in laboratory research [44], [45], [46], [47], [48], [60] suggest that body sway in non-provocative situations might be used to predict individual susceptibility to motion sickness [60]. Such predictive power, based on simple, non-invasive measures of objective behavior, could have significant practical value. Additional research is needed to determine whether a single pattern of sway precedes all forms of motion sickness, or whether different forms of motion sickness (e.g., seasickness, cybersickness, car sickness) are preceded by distinct patterns of body sway.

### Seasickness and mal de debarquement

Body sway differed between participants as a function of the severity of subsequent seasickness, but also as a function of the duration of subsequent mal de debarquement. These results are consistent with the hypothesis that unstable control of the body precedes all forms of motion sickness [53], [54]. Differences were observed in the spatial magnitude of body sway (in the case of mal de debarquement) but also in the temporal dynamics of sway, consistent with the hypothesis that the stability and instability of body sway cannot be defined exclusively in terms of the spatial magnitude of movement [47], [52], [53].

The severity of seasickness was correlated with the duration of mal de debarquement, replicating previous effects [65], [67]. Moreover, both seasickness and mal de debarquement were preceded by distinctive patterns of body sway. However, we found very different relations between body sway and each of these maladies. Seasickness was related only to patterns of body sway before the voyage, whereas mal de debarquement was related only to patterns of sway at sea. Moreover, the patterns of body sway that preceded the two maladies were not identical. For seasickness, the self-similarity of sway was directly related to the severity of symptoms, whereas for mal de debarquement the self-similarity of sway was inversely related to the duration of symptoms. In addition, seasickness was related only to the temporal dynamics of sway while mal de debarquement was related to both the temporal dynamics and the spatial magnitude of sway. These differences suggest that different forms of motion sickness may be preceded by distinct patterns of body sway.

### Multiple time scales in perceptual-motor adaptation

Our results suggest that different aspects of getting one's sea legs may have different time scales. Adjustments in body configuration and postural kinematics were complete within the first 24 hours of the voyage, but seasickness persisted. In these phenomena there may be a connection with the existence of different time scales for perceptual-motor learning [12], [71]. Further research is needed to understand relations between bodily control and the longer-term process of getting one's sea legs. To better understand the rapid changes in body configuration and postural control that we observed, earlier and more frequent sampling is needed [72]. In the broader context our results motivate increased attention to phenomena of perceptual-motor learning that take place over hours and days, such as learning to ride a bicycle, or perceptual-motor adaptation to prism spectacles.

It is likely that our measures of postural activity did not capture all aspects of perceptual-motor learning and adaptation that occurred in response to ship motion. We measured sway only once per day, in a narrow set of conditions, and with only two dependent variables. Other aspects of body sway may take longer to adapt to ship motion and, therefore, may have a time course more similar to that of seasickness. To fully understand temporal relations between body sway and seasickness it will be important to assess behavior over multiple time scales (e.g., minutes, hours, days), with frequent sampling (e.g., multiple testing sessions per day). Such an approach will permit us to observe the processes of change in individual behaviors, as well as relations between behaviors that change at different time scales [72].

### A model for aging and clinical conditions

The positional variability of body sway was greater at sea than before the voyage began. This change, which occurred “overnight”, resembles changes in standing body sway that, in other contexts, occur over much longer time scales. On land, healthy elderly adults tend to sway more than healthy young adults; this age effect has been observed in many studies across a wide variety of situations [73]. At sea, the body sway of our young adult participants resembled land-based measures of body sway in elderly adults. This resemblance applies also to the temporal dynamics of sway. In our young adult participants, the self-similarity of sway was reduced at sea, relative to sway when the ship was at the dock. The direction and magnitude of this decrease resembles differences in the temporal dynamics of sway between young and elderly adults on land [39]. Taken together, these results suggest that the relatively rapid effects of ship motion on postural control in young adults may offer a valid model for much more gradual effects of aging and pathology on postural control. As one example, getting one's sea legs may include exploration for and adoption of a new level of optimal variability [52]. This process, with a time scale measured in hours, may be a useful model for adjustments in movement variability that occur with time scales measured in months, such as pregnancy [74], or with time scales measured in years, such as aging [50], [52]. Getting ones' sea legs occurs in otherwise healthy persons, and is temporary, suggesting that healthy persons at sea might be useful and convenient models for a variety of clinical conditions. Our results suggest that it may be useful, in future studies, to examine relations between stance at sea and stance in clinical populations in the context of different parameters of stance, such as patterns of hip-ankle coordination [75]. Research of this kind can help to determine the extent to which stance at sea may be a useful model for stance in clinical populations.

## Conclusion

We conducted the first experimental evaluations of postural and subjective phenomena of getting one's sea legs. We documented a variety of effects that, taken together, illustrate the powerful, pervasive, and persistent influence that vehicle motion can have on the control of the body and on related subjective experience. Our results suggest that the visible horizon plays an important role in the stabilization of the body in any form of vehicular travel. In addition, our results suggest that the process of getting one's sea legs may help to illuminate phenomena of perceptual-motor adaptation on land, as well as at sea.

Our experiments constitute the first attempt to relate postural activity to the phenomena of getting one's sea legs (cf. [12]). Adaption to life at sea occurs over hours and days and, as such offers a venue for the study of adaptation in motor behavior over relatively long time scales. More broadly, research on the process by which people get their sea legs offers an opportunity to study natural adaptation of motor behavior in healthy adults. Descriptions of this natural adaptive process can help to constrain theories of perceptual-motor adaptation, can guide the development of clinical interventions, and can motivate new research.
